# A new leafhopper species of the genus *Anagonalia* from India (Hemiptera, Cicadellidae, Cicadellinae)

**DOI:** 10.3897/zookeys.1004.26253

**Published:** 2020-12-17

**Authors:** Stuti Rai, Naresh M. Meshram

**Affiliations:** 1 Division of Entomology, Indian Council of Agricultural Research-Indian Agricultural Research Institute, New Delhi 110012, India Indian Agricultural Research Institute New Delhi India

**Keywords:** Auchenorrhyncha, intra-specific variation

## Abstract

A new leafhopper species, *Anagonalia lapnanensis***sp. nov.**, is described from Arunachal Pradesh, India. A morphological variant is also described which, is interpreted as belonging to the same species due to negligible divergence in the COI mtDNA gene. Detailed illustration of males and female are provided.

## Introduction

**Figures 1–11.**
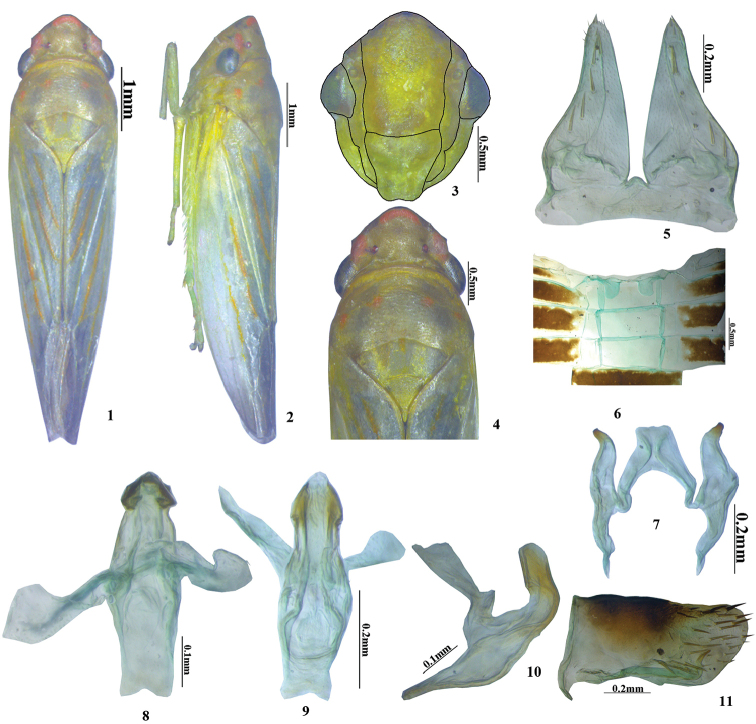
*Anagonalia lapnanensis* sp. nov. (male): **1** dorsal habitus **2** lateral habitus **3** face **4** head and thorax, dorsal view **5** subgenital plates and valve **6** sternal abdominal apodemes **7** styles and connective **8–10** aedeagus and dorsal connective, dorsal, ventral and lateral views **11** pygofer lateral view.

Members of the Asian leafhopper genus *Anagonalia* Young (Cicadellidae: Cicadellinae) are medium to large in size and either pale yellow to green with or without dark wings and usually with orange markings on the head and thorax (Fig. 1). Young (1986) established the genus *Anagonalia* with *Tettigoniella melichari* Distant from Sri Lanka (new name for *T. rubromaculata* Melichar, preoccupied) as the type species. Young (1986) also included two new species from south India, *A. koda* and *A. trava*, and synonymised *Tettigoniella affinis* Distant from north-east India with *A. melichari*. In addition to colour, the genus *Anagonalia* can be distinguished by its angulate anterolateral margin of the crown near the eye, relatively flattened clypeus, and depressed clypellus (Figs 3, 21). According to Young (1986) the last two features separate *Anagonalia* from the closely related *Erragonalia* Young but the depression of the clypellus (best seen in lateral view) is very slight in some species of *Anagonalia*. Young also mentioned that the genus differs in lacking basiventral microsetae on the pygofer but these are present in *A. melichari* sensu Yang et al. (2017: fig. 6a). In the present study we describe a new species from north-eastern region of India, together with a variant, and provide illustrations of external features and male and female genitalia.

## Materials and methods

Type specimens of the new species are deposited in National Pusa Collection, Division of Entomology, ICAR-Indian Agricultural Research Institute, New Delhi, India. Specimens were collected through Mercury vapour lamp light trap from Lapnan (India: Arunachal Pradesh), were processed by sorting, cleaning and mounting. Male genitalia dissection was carried out as described by Knight (1965). The abdomen was removed by inserting a sharp pin between the abdomen and thorax with gentle piercing. The abdomen was treated in 10% KOH for 2–4 h to remove unsclerotised material by gently prodding the abdomen with the head of a pin. Afterwards, the abdomen was rinsed thoroughly in water. The internal structures were then removed by a hooked pin, before being stored in glycerol vials for study. Photographs were taken with a Leica DFC 425C digital camera on the Leica M205FA stereozoom automontage microscope.

### Molecular analysis using DNA extraction and PCR amplification

For mitochondrial cytochrome oxidase subunit I (mtCOI) analysis, the DNA was extracted from legs of specimens according to the manufacturer protocol, QIAGEN QIAamp DNA Investigator Kit. The isolated DNA was stored at -20 °C until required. The barcode region of mtCOI was amplified using the primers LCO1490: 5’-GGTCAACAAATCATAAAGATATTGG-3’; HCO2198:5’-TAAACTTCAGGGTGACCAAAAAATCA-3’. The PCR was performed with total reaction volume of 25 μl using DNA polymerase (FermentasGmBH, St. Leon- Rot, Germany) under the following cycling protocol: 4 min. hot start at 94°, 35 cycles of denaturation for 30 s at 94°, annealing for 60 s at 47 °C, elongation for 50 s at 72 °C and a final extension 72 °C for 8 min in a C1000 Thermal cycler. The reaction mixture included (as described by KOD FX puregene™ manufacturer protocol) 4 μl of DNA template, 12.5 μl 2× PCR buffer, 10 μl 2mM dNTP, 1 unit TAQ (KODFX) enzyme, and forward and reverse primers were 0.3 μM each at final concentration. The products were checked on a 2% agarose gel and visualised under UV using Alphaview® software version1.2.0.1. The amplified products were sequenced at SciGenomePvt Ltd (Cochin, India). The sequences were assembled and aligned with BioEdit version 7.0.0 and deposited in NCBI GenBank (Table 1).

### Phylogenetic analysis

To determine the sequence divergence between the two variants of *A. lapnanensis* sp. n., we performed a phylogenetic analysis including other taxa within Cicadellini and Opsiini as outgroups (Table 1). The Basic Local Alignment Search Tool (BLAST) was used to query the National Center for Biotechnology Information (NCBI) non-redundant nucleotide database and protein database for other leafhopper COI sequence data in blastn searches. The sequences were aligned with the help of CLUSTAL W and a neighbour-joining tree was prepared with MEGA version 6 using Kimura-2-Parameter distance model (Tamura et al. 2013, Saitou and Nei 1987, Kimura 1980).

**Table 1.** Taxa included in phylogenetic analysis with GenBank accession numbers, with locality data.

Species	Geographic locality	GenBank sequence ID
*Anagonalia lapnanensis* sp. nov.	India: Arunachal Pradesh	MK073117*
*Anagonalia lapnanensis* variant	India: Arunachal Pradesh	MK073118*
*Atkinsoniella sulphurata*	China	HQ456375.1
*Atkinsoniella opponens*	China	HQ456374.1
*Atkinsoniella thalia*	China	HQ456372.1
*Atkinsoniella grahami*	China	HQ456377.1
*Hishimonus phycitis*	Not Available	KY654344.1

*Obtained from molecular work analysed for this paper

#### Anagonalia lapnanensis sp. nov.

Taxon classificationAnimaliaHemipteraCicadellidae

3D9DBADF-2939-5323-8941-4C1E219CF1DA

http://zoobank.org/02BF2478-0383-4C98-8C57-1C6EDEEE553A

Figs 1–11 , 12–18

##### Material examined.

***Holotype*** ♂, India: Arunachal Pradesh: Lapnan, (26°59'27.03"N, 95°28'58.29"E, 448m), 27.xi.2017, sweep net coll. Stuti (National Pusa Collection). ***Paratypes*** 5♀, same data as holotype.

##### Diagnosis.

The new species can be distinguished from other species of the genus in having pygofer process apically sinuate, short styles with indistinct lateral lobe, connective with short stem and strongly divergent arms, strongly bent aedeagal shaft with rectilinear apical part, and dorsal connective elongate and tapered distally.

##### Description.

Colour (Figs 1–4) generally pale green with yellow tinge. Head with three orange spots on crown, anterior spot saddle shaped, lateral pair elliptical, and one median translucent black spot on anterior margin in ventral view. Pronotum with four orange spots approximately equidistant from anterior and posterior margin and to each other. Scutellum medially with an orange spot and yellow mottling at anterior margin. Forewing with prominent orange markings on veins.

Head with anterior margin of crown rounded, anterolateral margin of crown angulate before eyes; crown longer medially than next to eyes, length medially approximately half width across eyes; ocelli on crown, almost equidistant from anterior and posterior margin, nearer to the lateral orange spots than to each other; ridge arising from eye up to ocelli, crown convex between ocelli. Face with frontoclypeus broad and relatively flat, laterofrontal suture extending onto crown; clypellus depressed distally (Fig. 3). Pronotum (Fig. 4) convex, posterior margin highly concave in middle, 1.3× wider than long and 1.22× longer than vertex.

Male second sternal abdominal apodemes (Fig. 9) short, not extending to next sternite.

*Male genitalia*. Pygofer (Fig. 11) in lateral view, 1.6× longer than wide, with dispersed macrosetae on apical half; caudoventral margin with row of stout macrosetae; in ventral view, a long apically sinuate hook like process arising on posteroventral margin. Valve short, fused to pygofer. Subgenital plates (Fig. 5) 1.4× as long as style, elongate, triangular, narrowed gradually towards apex, apex rounded, with a row of macrosetae along sub-lateral margin. Styles (Fig. 7) longer than wide, exceeding apex of connective, preapical lobe poorly developed.Connective (Fig. 7) Y-shaped with stem 1.5× longer than arms. Dorsal connective with arms tapered to apex. Aedeagus in dorsal view (Fig. 8), wider in the middle and slightly narrow at base and apex, in lateral view (Fig. 10) shaft strongly dorsally curved at midlength, of similar width throughout length, gonopore apical.

**Figures 12–18.**
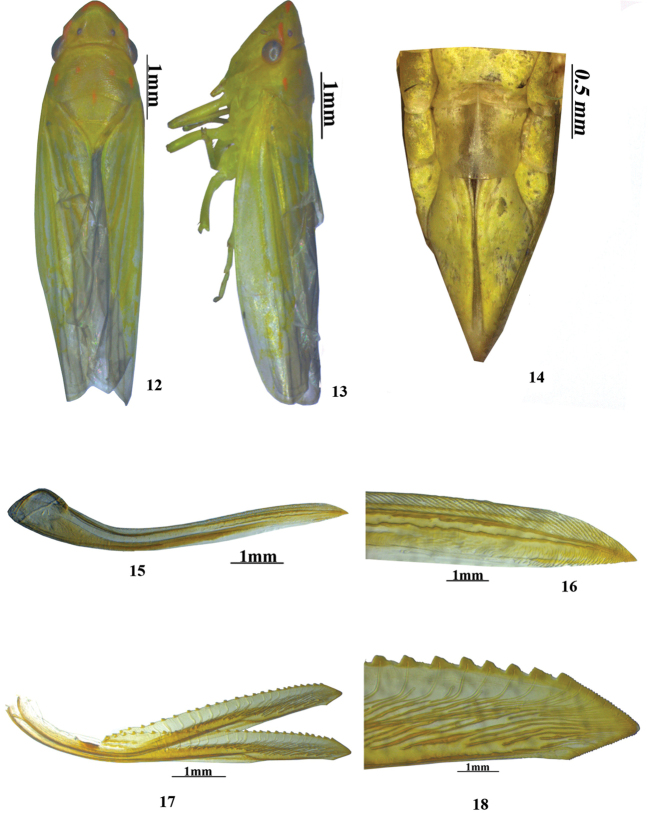
*Anagonalia lapnanensis* (female): **12** dorsal habitus **13** lateral habitus **14** sternite VII **15, 16** first valvula **17, 18** second valvula.

*Female genitalia*. Seventh sternite (Fig. 14) with posterior margin slightly convex; first valvulae (Figs 15, 16) slightly bent dorsally and tapered distally to acute apex; in lateral view dorsal irregular sculpturing extending from midlength to apex, subapically with ventral hyaline area extending to midlength. Second valvulae (Fig. 17) with dorsal sclerotised and hyaline area at ¼^th^ distance from base to apex, thereafter dorsal margin sharply expanded and slightly tapered to near apex with approximately 26 teeth, thereafter apex triangular with fine teeth on dorsal and ventral margins (Fig. 18).

Measurements (mm). Male 6.8 long, 1.5 wide across eyes, 1.4 wide across hind margin of pronotum. Female 6.3 long, 1.3 wide across eyes, 1.2 wide across hind margin of pronotum.

##### Etymology.

This species is named after the place of collection, Lapnan, in Arunachal Pradesh.

##### Remarks.

The new species is similar externally to the types of *Tettigoniella affinis* Distant from India, a species synonymised with *A. melichari* Distant from Sri Lanka by Young (1986) but, as the types of *T. affinis* are female, their identity is uncertain (MD Webb, pers. comm.). Based on figures seen of the types of *A. melichari* (Melanovsky & Webb, in prep.), the specimens described here differ in shape of the male genitalia by the pygofer process being strongly sinuate distally in lateral view, the aedeagal shaft apex being rectilinear in lateral view and the style lacking a preapical lobe. The specimen recorded by Young (1986) of *A. melichari* is figured in insufficient detail to be certain of its identity.

### Variant of the new species

**Material examined.** Non-types 2♂, same data as holotype and paratypes, except 10.vii.2018.

**Remarks.** This variant differs from the holotype and paratypes of the new species as follows: black with orange markings on head and thorax more prominent; scutellum with three spots, one medial and two lateral; crown less produced anteriorly; aedeagus in dorsal view narrower at apex and in lateral view apex wider subapically (see Figs 19–29).

**Figures 19–29.**
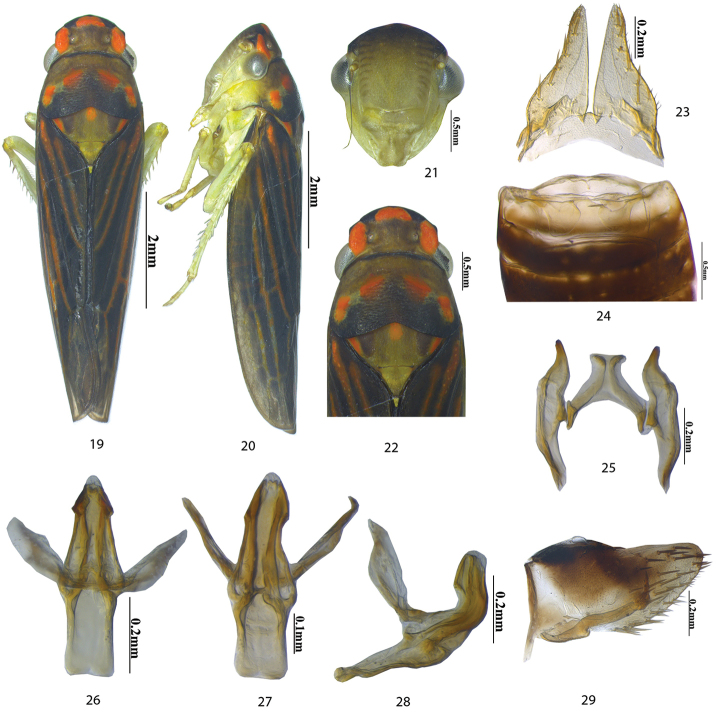
*Anagonalia lapnanensis* variant: **19** dorsal habitus **20** lateral habitus **21** face **22** head and thorax, dorsal view **23** subgenital plates and valve **24** sternal abdominal apodemes **25** styles and connective **26–28** aedeagus and dorsal connective, dorsal, ventral, and lateral views **29** pygofer, lateral view.

**Figure 30.**
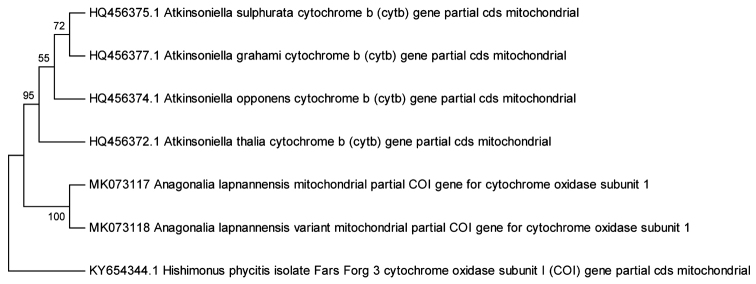
Phylogram showing relationships of *Anagonalia lapnanensis* sp. nov. and *A. lapnanensis* variant, with related species of tribe Cicadellini inferred using by neighbour-joining (NJ) tree method of mitochondrial COI sequences.

To establish if the variant was a new species a molecular analysis was performed (see Materials and methods). The neighbour-joining (NJ) tree (Fig. 30) from the phylogenetic analysis shows 100 % similarity and the Pairwise Distance matrix (Table 2) had shown genetic distance 0.2 %, which is negligible according to the 3.5 % divergence rule (De Barro et al. 2011). Thus it is inferred that the differences found in topotypical specimens are deemed to be variations of the new species rather than being of a different species. This variation may be attributed to seasonal climatic differences, i.e., the rainy season in July, and winter in November. Further confirmation of these findings will require breeding experiments and or acoustic recordings.

**Table 2.** Percent pairwise corrected (K2P) genetic distance among different species of Cicadellini including the new species and the variant for MtCOI.

Species	1	2	3	4	5	6	7
*Anagonalia lapnanensis* sp. nov.		0.002	0.074	0.078	0.081	0.076	0.141
*Anagonalia lapnanensis* variant	0.002		0.073	0.077	0.081	0.075	0.140
*Atkinsoniella sulphurata*	0.831	0.824		0.017	0.023	0.017	0.113
*Atkinsoniella opponens*	0.864	0.857	0.131		0.022	0.018	0.118
*Atkinsoniella thalia*	0.886	0.879	0.195	0.175		0.024	0.159
*Atkinsoniella grahami*	0.858	0.851	0.119	0.134	0.202		0.129
*Hishimonus phycitis*	1.329	1.328	1.141	1.151	1.319	1.248	

## Supplementary Material

XML Treatment for Anagonalia lapnanensis
